# Sleep misperception and associated factors among middle-aged women in Korea: a cross-sectional study

**DOI:** 10.7586/jkbns.25.032

**Published:** 2025-08-26

**Authors:** Gahye Kim, Meejung Chin, Sowon Hahn, Yeon Soo Kim, Young Hye Kwon, Yeon-Hwan Park

**Affiliations:** 1 College of Nursing, Eulji University, Uijeongbu, Korea; 2 Department of Child Development and Family Studies, Seoul National University, Seoul, Korea; 3 Department of Psychology, Seoul National University, Seoul, Korea; 4 Department of Physical Education, College of Education, Seoul National University, Seoul, Korea; 5 Department of Food and Nutrition, Seoul National University, Seoul, Korea; 6 College of Nursing, The Research Institute of Nursing Science, Seoul National University, Seoul, Korea

**Keywords:** Menopause, Middle aged, Sleep wake disorders, Wearable electronic devices

## Abstract

**Purpose:**

This study aimed to examine sleep misperception and identify its related factors in middle‑aged women.

**Methods:**

This descriptive cross-sectional study included 93 women aged 45~55 years. On the first day, participants completed self-report questionnaires assessing subjective sleep, demographic, menopausal, psychosocial, and lifestyle characteristics. They also underwent physical assessments, including grip strength and waist-to-height ratio. Objective sleep and physical activity were monitored over seven days using a Fitbit Charge 5. Sleep misperception was calculated using the Misperception Index. Discrepancies between objective and subjective sleep were assessed using the paired t-test and Bland-Altman plots. Influencing factors were identified via hierarchical multiple linear regression.

**Results:**

The mean value of the Misperception Index was 0.02 ± 0.18 (range: −0.55~0.52). Significant discrepancies were found between the objective and subjective measures for total time in bed and sleep efficiency, but not for total sleep time. Most values fell within the acceptable limits of agreement, although individual variability existed. Hierarchical regression showed that psychological menopausal symptoms (β = .31, *p* = .007), prolonged rapid eye movement sleep (β = .29, *p* = .003), and increased light sleep (β = .44, *p* < .001) were significant predictors of greater sleep misperception.

**Conclusion:**

This study highlights the multifactorial nature of sleep misperception in middle-aged women, which is influenced by psychological menopausal symptoms and sleep architecture. A multimodal approach integrating objective and subjective sleep measures with psychosocial evaluation is essential for developing and tailoring interventions to support sleep health.

## INTRODUCTION

Sleep health is crucial for overall well-being, particularly for middle-aged women undergoing significant hormonal transitions [1]. Although the definition of middle age varies across studies, the period comprising the ages of 45 to 55 years represents a pivotal transitional phase for women. It is marked by profound physical and psychosocial changes, including the onset and progression of menopause, shifts in family and work roles, and increased vulnerability to chronic health conditions [2-4]. This stage is characterized by fluctuations in reproductive hormones as well as by heightened psychosocial stressors, such as caregiving responsibilities for both aging parents and children transitioning to adulthood, career demands, and changes in social support networks [3]. Collectively, these factors may adversely affect women’s sleep quality and overall health outcomes.

Research suggests that 46.5% to 54.7% of menopausal women experience clinically substantial sleep disturbances [5]. The Study of Women's Health Across the Nation has demonstrated that fluctuations in follicle-stimulating hormone and estradiol levels during menopause are linked to nocturnal awakenings, as well as difficulties in sleep initiation and maintenance, with vasomotor symptoms and mood disturbances further exacerbating these sleep issues [4,6].

Sleep misperception, which refers to the discrepancy between objective and subjective sleep assessments, is a critical concern in sleep health management [7]. Underestimating sleep duration or quality may lead to the unnecessary use of sleep aids, increasing the risk of dependency and adverse effects [8]. Prolonged sleep underestimation can eventually lead to objective sleep disorders, including sleep deprivation [9]. Overestimation of sleep can mask underlying sleep disorders and delay timely diagnosis and intervention. A longitudinal study showed that the overestimation of sleep duration was to a decrease in rapid eye movement (REM) sleep and a higher risk of all-cause mortality, emphasizing the potential long-term health risks associated with this issue [10]. Therefore, thorough evaluation and targeted interventions for sleep misperception are paramount to prevent long-term sleep pathology and its associated health risks.

Although previous studies have explored sleep misperception-related factors, including demographics (sex, age, ethnicity, marital status, education, and socioeconomic status) [11-13], psychosocial variables (anxiety, depression, and social relationships) [11,12], and objective sleep parameters (wake after sleep onset, total sleep time, and sleep efficiency) [13,14], significant gaps persist in understanding this phenomenon in midlife women. Middle-aged women undergo physical and psychosocial changes associated with menopause, making it crucial to identify the factors, that influence sleep misperception. Although some studies have explored sex differences in middle-aged adults, in-depth investigations specifically targeting middle-aged women are lacking [12]. Existing research has predominantly focused on clinical populations, such as individuals with insomnia or depression [11,14,15], limiting the generalizability of the findings to community-dwelling middle-aged women. Many studies have used polysomnography (PSG) as the gold standard for objectively assessing sleep misperception [11,16]. However, this method has limitations, as it requires sleeping in an unfamiliar laboratory setting for a single night, which may not accurately reflect an individual's typical sleep patterns at home. While some researchers have employed wearable devices, such as actigraphy and accelerometers, to assess sleep in natural settings [12-14], these studies often lack a comprehensive consideration of factors closely related to sleep, including physical activity, specific sleep stages, and menopausal symptoms in midlife women. To address these limitations, the present study employed wearable devices to objectively measure sleep over multiple consecutive days in the participants’ usual home environments. By conducting multiday assessments in real-life settings, our study aimed to provide a more accurate and ecologically valid understanding of sleep misperception and its associated factors among community-dwelling middle-aged women.

## METHODS

### 1. Study design

This study used a descriptive cross-sectional design to explore sleep misperception and its associated factors in middle-aged women.

### 2. Participants

We recruited 100 participants through convenience sampling between February 14 and March 22, 2023. The sample size was determined using G*Power 3.1.9.7 software and was based on practical considerations, such as the availability of wearable devices. Recruitment notices were distributed to local government offices, public health centers, and the local online communities in Seoul, South Korea. The inclusion criteria were as follows: (a) women aged 45~55 years; (b) Android smartphone users—selected to ensure a consistent procedure for sleep data collection; and (c) those able to visit our laboratory for physical fitness assessment, self-report questionnaires, and wearable device setup. Participants who regularly took sleep medications were excluded. In addition, daily measurements based on wearable devices were excluded if the device wear time was less than 1,000 minutes, or if the most extended nighttime sleep period starting between 8:00 p.m. and 4:00 a.m. the following day was either less than 3 hours or more than 12 hours. Only the data from participants with at least four valid days were included in the analysis.

Of the 100 participants, two were outside the age range, three were taking regular sleep medication, and two did not meet the valid wear time criteria for the wearable device. A total of 93 participants were included in the analysis. The sample size was calculated using G*Power 3.1.9.7 software [17] with an alpha of .05, power of .80, medium effect size (f^2^ = .2), and 10 predictor variables [18]. The analysis indicated that a minimum of 91 participants were required for multiple regression analysis. Therefore, the final sample size of 93 participants was sufficient for this study.

### 3. Instruments

Data were obtained from objective assessments (objective sleep and physical activity, grip strength, waist-to-height ratio) and subjective self-report questionnaires (subjective sleep, demographic, menopause-related, psychosocial, and lifestyle characteristics excluding physical activity).

#### 1) Sleep parameters

Sleep parameters were assessed using both objective and subjective methods.

##### (1) Objective sleep

Objective sleep parameters were measured using the Fitbit Charge 5 (Fitbit Inc., San Francisco, CA, USA), a wearable device that evaluates sleep stages based on heart rate variability and body movement. This device was selected for its high sensitivity and specificity in detecting sleep stages and its demonstrated accuracy in measuring total sleep time (TST), wake after sleep onset (WASO), and sleep efficiency (SE), with no significant differences compared to PSG [19]. The objective sleep parameters used for data analysis included estimated total time in bed (TIB), TST, SE, WASO, and sleep stage duration (REM, light, and deep sleep).

##### (2) Subjective sleep

Subjective sleep parameters were assessed using self-report questionnaires. The questionnaire included open-ended questions regarding participants’ estimated TIB and TST in the previous month. SE was calculated by dividing TST by TIB.

##### (3) Sleep misperception

Sleep misperception was assessed using the Misperception Index (MI), calculated as MI = (objective TST-subjective TST)/objective TST [20]. For the calculation of MI, the objective TST was averaged over a 7-day measurement period. A positive MI value indicates underestimation of sleep duration, whereas a negative MI value suggests overestimation [21].

#### 2) Demographic characteristics

Demographic characteristics, including age, educational level, employment status, and economic status were assessed using a self-report questionnaire. Economic status was assessed in terms of monthly household income, which was categorized into three groups (< 6 million South Korean won [KRW], 6~9 million KRW, and ≥ 9 million KRW). These categories consider both the average monthly household income distribution from the Korean Household Income and Expenditure Survey [22] and within our study sample.

#### 3) Physical characteristics

Muscle strength was assessed by grip strength, measured objectively using a Takei dynamometer (T.K.K 5401, Takei Scientific Instruments Co., Ltd., Tokyo, Japan). Participants stood with their feet shoulder-width apart, and their grip strength was measured twice for each hand. The mean value was used to calculate the absolute grip strength (kg), which was then divided by body weight to obtain the relative grip strength for analysis.

Based on previous studies indicating an association between abdominal obesity and sleep quality [23], abdominal obesity was assessed using the waist-to-height ratio (WHtR), which was calculated by dividing waist circumference by height. A WHtR of 0.5 or greater was used to define abdominal obesity.

#### 4) Menopause-related characteristics

Participants completed a questionnaire assessing menopausal status (perimenopausal or postmenopausal) and menopausal symptoms. The severity of menopausal symptoms was measured using the Menopause Rating Scale (MRS), a validated instrument designed to quantify aging- and menopause-related complaints in women [24]. The MRS consists of 11 items across three subscales: somatic symptoms (four items: hot flushes/night sweats, heart discomfort, sleep problems, and muscle and joint problems), psychological symptoms (four items: anxiety, depressive mood, irritability, and exhaustion), and urogenital symptoms (three items: sexual problems, urinary problems, and vaginal dryness). Each item is rated on a 5-point Likert scale ranging from 0 (not present) to 4 (very severe), with higher scores indicating more severe menopausal symptoms.

Considering its potential multicollinearity with the dependent variable, one item related to sleep disturbance originally included in the somatic subscale was excluded from the analysis. The total score ranges from 0 to 40, with subscale scores ranging from 0 to 16 for psychological domains and 0 to 12 for both the somatic and urogenital domains. In the original validation study, the internal consistency of the MRS was acceptable with a Cronbach’s alpha of .83.

#### 5) Psychosocial characteristics

Loneliness and social isolation were assessed using the Loneliness and Social Isolation Scale [25], a self-report questionnaire consisting of six items rated on a 4-point Likert scale and including three subscales: loneliness (two items), social support (two items), and social network (two items). A high risk of loneliness is defined as a score ≥ 3 on the loneliness subscale, while a high risk of social isolation is defined as scores ≥ 4 on both the social support and social network subscales [25]. The internal consistency of the original scale was acceptable (Cronbach’s α = .77).

#### 6) Lifestyle characteristics

Lifestyle characteristics included smoking status, alcohol and caffeine consumption, all of which were self-reported. Smoking status and alcohol consumption were assessed dichotomously (yes or no). Caffeine consumption was categorized as regular or occasional, with regular consumption defined as the consumption of five or more caffeinated beverages (e.g., coffee and tea) per week. Physical activity was measured objectively using a Fitbit Charge 5, recording daily steps, light physical activity (LPA), moderate physical activity, and vigorous physical activity (VPA) in minutes per day, and moderate-to-VPA (MVPA) in minutes per week.

### 4. Data collection

The participants visited the laboratory for anthropometric and physical fitness assessments to measure physical variables. The participants also completed self-administered questionnaires to collect information on subjective sleep parameters, demographic, menopausal, psychosocial, and lifestyle characteristics. Following these assessments, each participant received a Fitbit Charge 5 that was synchronized with their smartphone. Participants were instructed to wear the device on their non-dominant wrist for seven consecutive days, except during charging, showering, or activities such as dishwashing. Daily reminder messages were sent to encourage continuous wear and device synchronization. After the monitoring period, the participants returned to the laboratory, logged into a Fitbit-linked website, and downloaded their sleep and physical activity data as Excel files for analysis.

### 5. Data analysis

Paired t-tests were conducted to assess the differences between the objective and subjective sleep parameters. The discrepancy between the objective and subjective measurements was evaluated using Bland-Altman plots, which provided a visual representation of the mean difference and 95% limits of agreement (LoA) between the two measurement methods. Differences in sleep misperception subtypes according to participant characteristics were analyzed using the chi-square test for categorical variables and the independent t-test for continuous variables. Factors influencing sleep misperception were examined using hierarchical multiple linear regression. In the regression analysis, daily step counts were converted to units of 1,000 steps to prevent confusion in the interpretation of regression coefficients due to differences in variable scales. Variables with a *p*-value < .05 in univariate analyses were included in the regression model. The missing values were imputed using a regression imputation method. Data analysis was performed using SPSS version 23.0 (IBM Corp., Armonk, NY, USA).

### 6. Ethical considerations

This study was approved by the Institutional Review Board of Seoul National University (approval number: 2302/002-008). After the purpose and requirements of the study were fully explained, all participants voluntarily provided written informed consent before participating.

## RESULTS

### 1. General characteristics of the participants

Table 1 shows the participants’ demographic characteristics and study variables. The mean age of the participants was 50.24 years, and approximately two-thirds had a bachelor's degree or higher. Of the participants, 45.2% were post-menopausal. The mean scores for menopausal symptoms were somatic symptoms (2.89 out of 12), psychological symptoms (4.71 out of 16), and urogenital symptoms (3.57 out of 12). Less than 30% of the participants were classified as having a high risk of loneliness, whereas only 2.2% were categorized as having a high risk of social isolation. Most participants were non-smokers and regular caffeine consumers. Participants walked an average of more than 10,000 steps per day and engaged in an average of 341.32 minutes of MVPA per week. On average, participants spent 80.22 ± 18.36 minutes per day in REM sleep and 237.40 ± 32.10 minutes per day in light sleep.

### 2. Discrepancy between objective and subjective sleep parameters

A statistically significant difference was observed for TIB (*p* < .001), with objective measures (437.15 min) being significantly higher than subjective reports (406.72 min). While objective TST (378.43 min) was slightly higher than that of the subjective reports (367.47 min), this difference was not statistically significant (*p* = .128). The subjective SE (90.8%) was significantly higher than the objective SE (86.7%) (*p* = .002) (Table 2).

**Figure 1.**
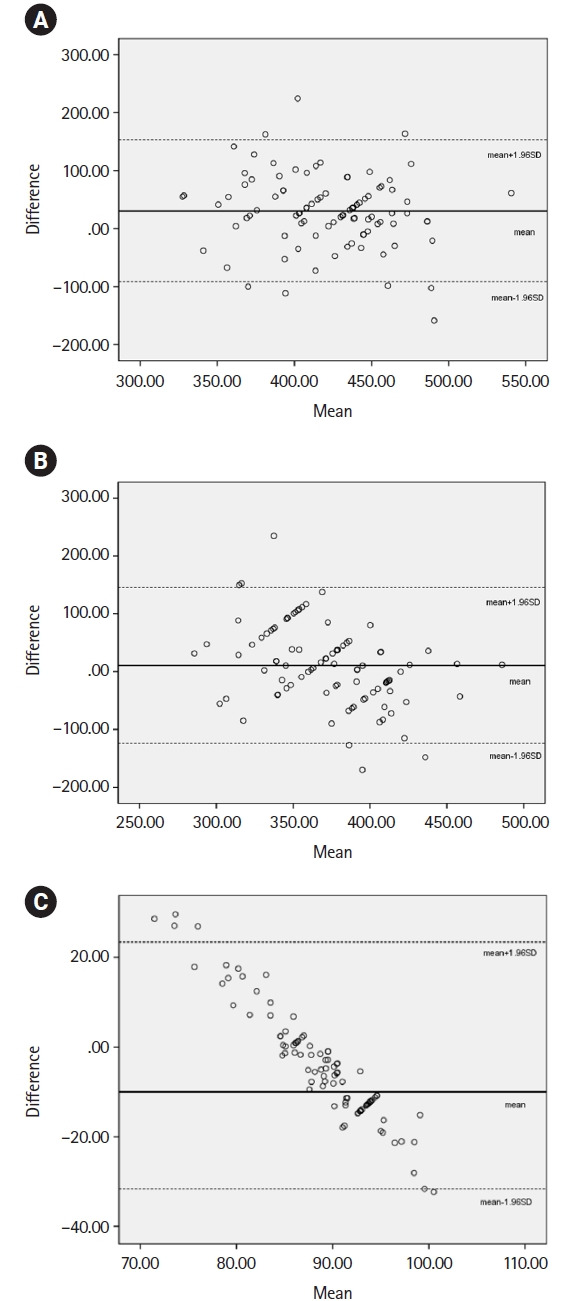
Bland-Altman plot illustrating the discrepancies between objective and subjective sleep parameters (A: total time in bed, B: total sleep time, and C: sleep efficiency) in middle-aged women (N = 93).

Bland-Altman analysis indicated some discrepancies between the objective and subjective sleep measurements (Figure 1). For TIB, the mean difference was 30.43 minutes, with 95% LoA ranging from −91.90 to +152.75 minutes, reflecting a tendency to underestimate TIB. Similarly, TST showed a mean difference of 10.96 minutes (95% LoA: −124.02 to +145.93). In contrast, SE demonstrated an inverse pattern, with a mean bias of −4.11% (95% LoA: −31.7% to +23.5%), indicating an overestimation of sleep efficiency. Most data points fell within the agreement limits; however, notable outliers were observed for TIB (n = 8), TST (n = 6), and SE (n = 5), suggesting that individual variability may affect clinical interpretation. Additionally, SE showed a proportional bias by having a negative relationship between the differences and the mean of the measurements (Figure 1).

### 3. Comparison of characteristics by sleep misperception subtypes

The mean MI score was 0.02 ± 0.18 (range: −0.55 ~ 0.52). Of the 93 participants, 40 were classified as overestimators (MI < 0), and 53 as underestimators (MI > 0) of sleep time. As shown in Table 1, there were no significant group differences in demographic characteristicss. While total menopausal symptom scores did not differ significantly between groups (*p* = .322), the underestimation group reported significantly higher somatic symptoms (3.08 ± 2.05) compared to the overestimation group (2.65 ± 2.03; *p* = .022). Significant differences were found in lifestyle characteristics, specifically in physical activity. The overestimation group exhibited greater engagement in daily steps (t = 2.15, *p* = .034) and LPA (t = 2.42, *p* = .018) than the underestimation group (Table 1).

### 4. Factors associated with sleep misperception

Before conducting hierarchical multiple regression analyses, the variance inflation factors (VIFs) between the predictor variables were examined. The VIFs ranged from 1.41 to 2.00, all below the threshold of 10, indicating no concern regarding multicollinearity. In addition, the Durbin–Watson statistic was 1.89, suggesting no autocorrelation in the residuals.

Univariate analyses identified several variables significantly associated with sleep misperception, including somatic menopausal symptoms (F = 4.65, *p* = .034), psychological menopausal symptoms (F = 12.96, *p* = .001), daily steps (F = 4.74, *p* = .032), LPA (F = 5.63, *p* = .020), WASO (F = 5.00, *p* = .028), light sleep (F = 16.11, *p* < .001), and REM sleep (F = 8.71, *p* = .004). These variables were entered into a hierarchical regression analysis.

In Model 1, which included demographic characteristics, no significant predictors of sleep misperception were identified. Model 2, incorporating menopausal symptoms, explained 9.1% of the variance in sleep misperception. Psychological menopausal symptoms were significantly associated with sleep misperception (β = .38, *p* = .002). Upon adding lifestyle factors (daily steps and LPA) to the Model 3, an additional 1.9% of the variance was explained; however, lifestyle characteristics did not have a statistically significant association with sleep misperception. Psychological menopausal symptoms remained significantly associated with sleep misperception (β = .34, *p* = .008). The final model, which incorporated objective sleep parameters, explained 29.8% of the variance in sleep misperception and was highly significant (F = 4.25, *p* < .001). REM sleep (β = .29, *p* = .003) and light sleep (β = .44, *p* < .001) emerged as significant predictors, while psychological menopausal symptoms continued to show a significant association with sleep misperception (β = .31, *p* = .007) (Table 3).

## DISCUSSION

Our findings demonstrated an acceptable agreement between objective and subjective sleep measures in middle-aged women, with mean MI of 0.02 ± 0.18 (range: −0.55 ~ 0.52). This indicates that there were minor discrepancies between objective and subjective sleep durations for most participants, supporting the findings of previous research [26]. While there is no universally accepted cutoff for clinically significant sleep misperception, a previous study has suggested that an MI of 0.9 or higher may be a clinically relevant indication of insomnia risk [20]. None of the participants in our sample reached this threshold, most likely because of the inclusion criteria related to the use of sleep medication. Nonetheless, the observed range of MI values highlights the presence of both underestimated and overestimated sleep duration among middle-aged women. Furthermore, the Bland-Altman analysis revealed considerable individual differences in sleep perception among some participants. This finding supports previous research showing that subjective sleep duration cannot be reliably predicted using Fitbit data alone [26], highlighting the notable individual variability in sleep misperception. These results emphasize the limitations of relying solely on either objective or subjective measurements to assess sleep in middle-aged women and underscore the need for a more individualized approach.

Additionally, we observed a proportional bias in the Bland-Altman plot in the perception of SE, such that participants with a lower mean SE tended to exhibit a greater underestimation of their sleep quality. In other words, lower SE was associated with a greater likelihood of perceiving one’s sleep quality more negatively. This may be explained by the fact that reduced SE is often accompanied by frequent awakenings and rapid transitions between sleep stages, which can distort the perception of sleep continuity [27,28]. Future studies with larger sample sizes are required to investigate this trend further.

Psychological menopausal symptoms were significant predictors of sleep misperception, consistent with the findings of previous studies [29,30]. In a study of adults with insomnia, higher depressive symptoms were linked to greater sleep misperception, with depression mediating the relationship between sleep misperception and insomnia severity [29]. Additionally, a longitudinal study reported that anxiety in menopausal women was associated with increased sleep latency and early morning awakenings [30], which are indicators of subjective sleep disturbance. Considering the increased vulnerability to depression and anxiety during menopause [31], our study emphasizes the need for psychological assessments to evaluate sleep-related complaints among middle-aged women. Early identification of and timely intervention for psychological issues in this population can help prevent the progression of severe insomnia.

Previous studies demonstrated that somatic menopausal symptoms such as hot flashes and sweating are associated with poorer sleep quality in middle-aged women [2,32]. In our study, while somatic symptoms were more severe among women who underestimated their sleep than among those who overestimated it, regression analysis revealed that these symptoms did not independently predict sleep misperception; instead, psychological symptoms had a stronger direct effect. This suggests that, although somatic symptoms may contribute to group differences in sleep perception, psychological factors play a more critical role in sleep misperception among middle-aged women. Given the potential for somatic and psychological symptoms to interact with and exacerbate sleep issues [32], integrated clinical interventions addressing both symptom types should be considered. Furthermore, future research should clarify the complex mechanisms linking somatic and psychological symptoms to sleep misperception in this population.

Our results show that higher physical activity levels do not necessarily enhance the accuracy of sleep perception in middle-aged women. Although previous studies have documented the positive effects of physical activity on objective sleep parameters [33,34], including improved sleep quality and efficiency, these benefits do not consistently extend to subjective sleep assessments. Recent research supports this dissociation, showing that physical activity does not significantly affect perceived sleep duration or quality, suggesting that other factors may play a more substantial role in sleep misperception [35]. This underscores the fact that simply increasing physical activity may be insufficient to address the discrepancies between objective and subjective sleep in this population.

We found that longer durations of REM sleep and light sleep were associated with increased sleep misperception, particularly in the form of an underestimation of TST. This result is in line with a previous study that reported that sleep onset misperception was related to an increased percentage of non-REM stage 1 and WASO during the first sleep cycle [36]. These results may be explained by the fact that both light sleep and REM sleep are characterized by high-frequency electroencephalogram activity, which more closely resembles wakefulness than the deeper stages of sleep [37]. Our findings emphasize the importance of considering sleep architecture when evaluating subjective sleep complaints and suggest that interventions targeting only TST may not adequately address sleep misperceptions in middle-aged women.

This study had several limitations. First, its cross-sectional design precluded any inference of causality between sleep misperception and its associated factors. Longitudinal research is necessary to clarify the temporal relationships and potential causal mechanisms. Second, the study sample was limited to middle-aged women who visited our laboratory, which may have introduced selection bias and limited the generalizability of the results. Third, the sleep assessment period was relatively short (7 days), which may not fully capture the variability in habitual sleep patterns. However, this study collected sleep data from participants over multiple nights in their usual environments using Fitbit devices. Previous studies have demonstrated this approach to have moderate to high accuracy in detecting light sleep (69%~81%) and REM sleep (62%~89%) compared to PSG [19]. Hence, our study is considered to have greater ecological validity than single night laboratory sleep assessments.

Despite these limitations, our findings have important clinical implications for identifying modifiable factors of sleep misperception in middle-aged women. These findings highlight the potential of targeted non-pharmacological interventions, such as cognitive-behavioral therapy for insomnia and sleep hygiene education, to improve sleep perception and overall sleep health in this population. Given the complex interplay between psychological symptoms and sleep architecture, integrated clinical approaches that address both mental health and sleep structure are recommended. This study supports the prioritization of safe, effective, and sustainable behavioral therapies over pharmacological treatments as first-line options for managing sleep misperception in middle-aged women.

## CONCLUSION

This study emphasizes the complex and individualized nature of sleep misperception in middle-aged women. Psychological menopausal factors, such as depression and anxiety, along with changes in sleep architecture, including prolonged REM sleep and light sleep, play a significant role in sleep misperception. Given the observed individual variability, comprehensive evaluations that integrate both objective and subjective sleep measures along with psychological and sleep-stage assessments are essential for accurate diagnosis and tailored interventions. Addressing these multifactorial influences is crucial for improving sleep health and the quality of life in this population.

## Figures and Tables

**Table 1.** General Characteristics and Comparisons between Sleep Misperception Groups (N = 93)

Variables	Categories	Total	Overestimation (n = 40)	Underestimation (n = 53)	*t*/*χ*^2^	*p*
Demographic characteristics						
Age (year)		50.24 ± 3.14	50.05 ± 3.00	50.38 ± 3.25	−0.50	.621
Educational level	≤ Associate degree	29 (31.2)	10 (25.0)	19 (35.8)	1.25	.263
	≥ Bachelor's degree	64 (68.8)	30 (75.0)	34 (64.2)		
Employment status	Employed	54 (58.1)	23 (57.5)	31 (58.5)	0.01	.924
	Unemployed	39 (41.9)	17 (42.5)	22 (41.5)		
Monthly household income (KRW)	< 6 million	29 (31.2)	12 (30.0)	17 (32.1)	2.87	.238
	6~9 million	35 (37.6)	12 (30.0)	23 (43.4)		
	≥ 9 million	29 (31.2)	16 (40.0)	13 (24.5)		
Physical characteristics						
Relative grip strength		41.81 ± 7.89	42.85 ± 9.63	41.03 ± 6.24	1.11	.271
WHtR	Normal (< 0.5)	56 (60.2)	26 (65.0)	30 (56.6)	0.67	.413
	Abdominal obesity (≥ 0.5)	37 (39.8)	14 (35.0)	23 (43.4)		
Menopause-related characteristics						
Menopause status^†^	Premenopausal	50 (53.8)	22 (55.0)	28 (52.8)	0.12	.733
	Postmenopausal	42 (45.2)	17 (42.5)	25 (47.2)		
Menopausal symptoms	Total	12.79 ± 6.28	11.08 ± 6.66	14.08 ± 5.71	−1.0	.322
	Somatic	2.89 ± 2.04	2.65 ± 2.03	3.08 ± 2.05	−2.34	.022
	Psychological	4.71 ± 2.73	4.15 ± 2.77	5.14 ± 2.64	−1.75	.084
	Urogenital	3.57 ± 2.49	3.20 ± 2.66	3.85 ± 2.33	−1.25	.214
Psychosocial characteristics						
Loneliness	Non-risk group	69 (74.2)	30 (75.0)	39 (73.6)	0.02	.877
	High-risk group	24 (25.8)	10 (25.0)	14 (26.4)		
Social isolation^†^	Non-risk group	90 (96.8)	39 (97.5)	51 (96.2)	-	1.000^‡^
	High-risk group	2 (2.2)	1 (2.5)	1 (1.9)		
Lifestyle characteristics						
Smoking	No	91 (97.8)	39 (97.5)	52 (98.1)	-	.675^‡^
	Yes	2 (2.2)	1 (2.5)	1 (1.9)		
Alcohol consumption	No	40 (43.0)	16 (40.0)	24 (45.3)	0.26	.610
	Yes	53 (57.0)	24 (60.0)	29 (54.7)		
Caffeine consumption	Regular	72 (77.4)	31 (77.5)	41 (77.4)	-	.987
	Occasional	21 (22.6)	9 (22.5)	12 (22.6)		
Physical activity	Daily steps	11,033.83	12,139.64	10,199.262	2.15	.034
		± 4,392.37	± 5,049.53	± 3,655.11		
	LPA (minutes/day)	248.40 ± 62.65	266.02 ± 67.51	235.10 ± 55.74	2.42	.018
	MPA (minutes/day)	20.61 ± 17.88	23.10 ± 21.99	18.72 ± 13.96	1.10	.275
	VPA (minutes/day)	28.16 ± 21.57	30.34 ± 21.18	36.50 ± 21.92	0.85	.398
	MVPA (minutes/week)	341.32 ± 236.32	374.13 ± 261.04	316.57 ± 215.05	1.17	.247
Objective sleep parameters						
WASO (minutes/day)		58.71 ± 11.24	56.80 ± 11.21	60.16 ± 11.15	−1.44	.155
REM sleep (minutes/day)		80.22 ± 18.36	75.09 ± 21.19	84.10 ± 14.97	−2.40	.018
Light sleep (minutes/day)		237.40 ± 32.10	225.74 ± 28.63	246.20 ± 32.02	−3.19	.002
Deep sleep (minutes/day)		60.80 ± 12.12	61.21 ± 12.90	60.50 ± 11.62	0.28	.783

Values are presented as mean ± standard deviation or number (%). WHtR = Waist-to-height ratio; LPA = Low-intensity physical activity; MPA = Moderate-intensity physical activity; VPA = Vigorous-intensity physical activity; MVPA = Moderate-to-vigorous physical activity; WASO = Wake after sleep onset; REM = Rapid eye movement.

† One missing value;

‡ Fisher's exact test.

**Table 2.** Discrepancies between Objective and Subjective Sleep Parameters (N = 93)

Sleep parameter	Objective	Subjective	t	*p*
Total time in bed (minutes)	437.15 ± 47.05	406.72 ± 54.89	4.70	< .001
Total sleep time (minutes)	378.43 ± 39.79	367.47 ± 61.35	1.53	.128
Sleep efficiency (%)	86.7 ± 1.8	90.8 ± 12.1	−3.15	.002

Values are presented as mean ± standard deviation.

**Table 3.** Factors Associated with Sleep Misperception (N = 93)

Variables	Categories	Model 1			Model 2			Model 3			Model 4		
		*β*	t	*p*	*β*	t	*p*	*β*	t	*p*	*β*	t	*p*
Demographic characteristics													
Age (year)		.01	0.11	.912	−.03	−0.29	.772	−.01	−0.06	.956	< .01	.0.02	.984
Educational level (ref ≥ Bachelor's degree)	≤ Associate degree	.07	0.61	.545	.18	1.68	.096	.19	1.77	.081	.18	1.93	.057
Employment (ref employed)	Unemployed	.02	0.22	.825	.07	0.67	.503	.06	0.61	.541	.01	0.12	.905
Monthly household income (KRW) (ref ≥ 9 million)	< 6 million	.02	0.15	.880	−.01	−0.11	.913	< .01	< 0.01	.999	.01	0.04	.965
	6-9 million	.01	0.08	.940	.03	0.23	.816	.01	0.10	.920	.04	−0.40	.690
Menopause-related characteristics													
Menopausal symptoms	Somatic				.06	0.50	.616	.06	0.53	.600	.03	0.32	.751
	Psychological				.38	3.17	.002	.34	2.73	.008	.31	2.79	.007
Lifestyle characteristics													
Daily steps								−.13	−1.04	.301	−.05	−0.42	.677
LPA (minutes/day)								−.10	−0.83	.409	−.09	−0.78	.435
Objective sleep parameters													
WASO (minutes/day)											−.11	−0.92	.362
REM sleep (minutes/day)											.29	3.09	.003
Light sleep (minutes/day)											.44	3.72	< .001
F (*p*)		0.09 (.994)			2.31 (.033)			2.26 (.025)			4.25 (< .001)		
Adjusted R^2^		−.05			.09			.11			.30		

ref = Reference; LPA = Low-intensity physical activity; WASO = Wake after sleep onset; REM = Rapid eye movement.

## Data Availability

Please contact the corresponding author for data availability.
